# Lymphomonocytic Extracellular Vesicles Influence Fibroblast Proliferation and Collagen Production in Systemic Sclerosis

**DOI:** 10.3390/ijms26062699

**Published:** 2025-03-17

**Authors:** Giuseppe Argentino, Bianca Olivieri, Matteo Morandi, Giulio Bonisoli, Ruggero Beri, Elisa Tinazzi, Simonetta Friso

**Affiliations:** 1 Internal Medicine Unit B, Department of Medicine, University of Verona, 37134 Verona, Italy; giulioluigi.bonisoli@univr.it (G.B.); ruggero.beri@univr.it (R.B.); elisa.tinazzi@aovr.veneto.it (E.T.); simonetta.friso@univr.it (S.F.); 2 Allergy Unit and Asthma Center, Verona Integrated University Hospital, 37134 Verona, Italy; biancaolivieri92@gmail.com; 3 Internal Medicine Unit, Valli del Noce Hospital, APSS Trento, 38023 Cles, Italy; matteo.morandi@apss.tn.it

**Keywords:** systemic sclerosis, extracellular vesicles, fibroblasts, fibrosis, Jurkat, U937

## Abstract

Systemic sclerosis (SSc) is a chronic autoimmune disorder characterized by fibrosis, immune dysregulation, and vascular abnormalities. Extracellular vesicles (EVs), secreted by immune cells, have been implicated in modulating fibroblast activity and are actively involved in SSc pathogenesis. This study aims to determine whether lymphomonocytic-derived EVs influence fibroblast proliferation and collagen synthesis in SSc. Fibroblasts from healthy donors (HDFs) and SSc patients (SScHDFs) were exposed to EVs derived from Jurkat and U937 cell lines stimulated under pro-inflammatory conditions using tumor necrosis factor-α (TNFα) or phorbol 12-myristate 13-acetate + ionomycin (PMA + IONO). Proliferation was assessed using CCK-8 assays, while collagen production was quantified via ELISA. Our findings demonstrate that EVs derived from PMA + IONO-stimulated Jurkat and U937 cells significantly reduced fibroblast proliferation in a dose-dependent manner. Notably, SScHDFs exhibited lower baseline proliferation and a diminished overall response to EV treatment. Collagen production was markedly reduced in both fibroblast types following exposure to PMA + IONO-stimulated EVs, whereas TNFα-stimulated EVs affected only HDFs. These findings suggest that EVs from activated immune cells modulate fibroblast function in SSc, potentially contributing to disease pathogenesis. Further research is warranted to elucidate the molecular mechanisms underlying these effects and to explore the therapeutic potential of targeting EV-mediated signaling in SSc.

## 1. Introduction

Systemic sclerosis (SSc) is a rare connective tissue disorder characterized by multisystem involvement, significant clinical heterogeneity, and a chronic, often progressive course. The hallmark pathological features of SSc include immune dysregulation, widespread microangiopathy, and fibrosis affecting the skin and internal organs. Based on the extent of cutaneous involvement, SSc is classified into diffuse cutaneous SSc (dcSSc) and limited cutaneous SSc (lcSSc), each with distinct clinical and serological profiles [1].

Fibroblasts play a pivotal role in SSc pathogenesis by contributing to excessive extracellular matrix production and fibrosis, driven by aberrant activation and persistent stimulation by profibrotic cytokines such as transforming growth factor-β (TGFβ) [2]. Emerging evidence suggests that endothelial-to-mesenchymal transition (EndoMT) and oxidative stress further exacerbate fibroblast activation and fibrosis [3]. Additionally, inflammatory mediators such as tumor necrosis factor-α (TNFα) have been implicated in fibroblast activation and excessive collagen deposition, exacerbating fibrotic progression in SSc [4,5].

Extracellular vesicles (EVs) are membrane-bound particles released by various cell types that facilitate intercellular communication and immune regulation. Increasing evidence supports a critical role for EVs in autoimmune diseases, particularly SSc [6,7]. Studies have investigated endothelial cell-derived EVs in SSc [8,9,10], highlighting their potential as biomarkers of vascular damage and their modulation in response to therapeutic interventions such as Iloprost infusion [11]. Furthermore, EVs have been proposed as non-invasive biomarkers for the early detection of pulmonary arterial hypertension (PAH) in SSc, with elevated levels of platelet-derived EVs (CD42a) and immune-related markers (CD3 and CD56) indicating their involvement in disease progression and vascular dysfunction [12].

EVs transport bioactive molecules, including proteins, lipids, and RNAs, which modulate recipient cell behavior, including fibroblast activity [13]. Recent studies suggest that EVs facilitate the extension of fibrosis from affected to unaffected tissues via paracrine signaling, delivering profibrotic cargo such as TGFβ from SSc lung tissues and fibroblasts to recipient cells, thereby promoting myofibroblast conversion and fibrosis progression in vitro and in vivo [14,15]. Given their role in fibrotic signaling, EVs have garnered significant interest as potential biomarkers and therapeutic targets in SSc [6,16,17]. Of particular interest, mesenchymal stromal cell-derived EVs have emerged as a promising therapeutic approach, demonstrating antifibrotic and immunomodulatory effects in SSc, as evidenced in both in vitro and murine models [18,19,20,21].

This study aims to investigate the impact of lymphomonocytic-derived EVs on the proliferation and collagen production of fibroblasts from healthy individuals and SSc patients. Specifically, we examine the responses of HDFs and SScHDFs to EVs derived from Jurkat and U937 cell lines stimulated under pro-inflammatory conditions using TNFα or phorbol 12-myristate 13-acetate + ionomycin (PMA + IONO). By comparing fibroblast responses, we aim to elucidate the differential impact of EVs in SSc fibrosis, thereby providing insights into potential pathogenic mechanisms and therapeutic targets.

## 2. Results

### 2.1. Effects of Extracellular Vesicles on Fibroblast Proliferation

**Figure 1:**
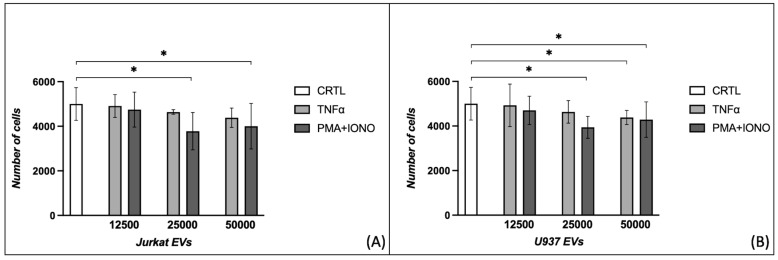
Evaluation of the effects of three different concentrations of extracellular vesicles on fibroblast proliferation. HDFs reduce proliferation when incubated with 25,000/50,000 EVs derived from (**A**) Jurkat or (**B**) U937 cells treated with PMA + IONO, as well as with 50,000 EVs obtained from U937 cells stimulated with TNFα (* *p* < 0.05). HDFs: human dermal fibroblasts; EVs: extracellular vesicles; PMA: phorbol 12-myristate 13-acetate; IONO: ionomycin; TNFα: tumor necrosis factor-alpha.

The proliferation of HDFs significantly decreased when incubated with 25,000 and 50,000 EVs from Jurkat cells stimulated with PMA + IONO (5000 ± 732 vs. 3779 ± 836, *p* = 0.013; 5000 ± 732 vs. 4003 ± 1022, *p* = 0.032) (Figure 1A). Additionally, 25,000 EVs from U937 cells stimulated with PMA + IONO reduced HDF proliferation (5000 ± 732 vs. 3941 ± 491, *p* = 0.013), while 50,000 EVs from U937 cells stimulated both with TNFα and PMA + IONO showed statistically significant effects (5000 ± 732 vs. 4384 ± 314, *p* = 0.047; 5000 ± 732 vs. 4290 ± 794, *p* = 0.047) (Figure 1B).

**Figure 2:**
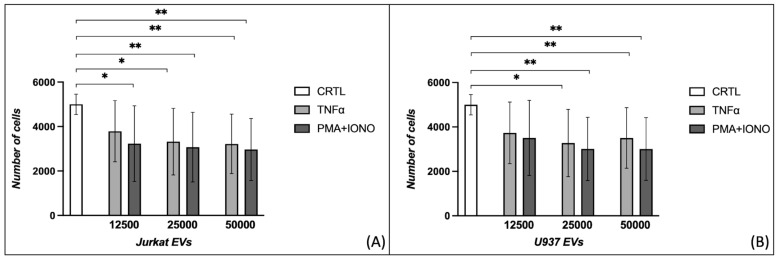
Evaluation of the effects of three different concentrations of extracellular vesicles on scleroderma fibroblast proliferation. SScHDFs reduce proliferation when incubated with (**A**) 12,500/25,000/50,000 EVs derived from Jurkat cells treated with PMA + IONO or 25,000/50,000 EVs treated with TNFα. (**B**) Incubation with 25,000/50,000 EVs from U937 cells reduces proliferation, and incubation with 50,000 EVs obtained from U937 cells stimulated both when incubated with PMA + IONO rather than TNFα (* *p* < 0.05, ** *p* < 0.005). SScHDFs: scleroderma human dermal fibroblasts; EVs: extracellular vesicles; PMA: phorbol 12-myristate 13-acetate; IONO: ionomycin; TNFα: tumor necrosis factor-alpha.

The scleroderma counterpart of dermal fibroblasts exhibited reduced proliferation in each condition of PMA + IONO EVs derived from Jurkat cells stimulated with PMA + IONO (5000 ± 457 vs. 3226 ± 1703, *p* = 0.047; 5000 ± 457 vs. 3075 ± 1567, *p* = 0.008; 5000 ± 457 vs. 2969 ± 1395, *p* = 0.003) and at the two highest concentrations of those stimulated with TNFα (5000 ± 457 vs. 3322 ± 1499, *p* = 0.021; 5000 ± 457 vs. 3218 ± 1337, *p* = 0.008) (Figure 2A). The concentrations of 25,000 and 50,000 EVs from U937 cells reduced the proliferation of SScHDFs when stimulated with both TNFα (5000 ± 457 vs. 3278 ± 1515, *p* = 0.021; 5000 ± 457 vs. 3505 ± 1360, *p* = 0.008) or PMA + IONO (5000 ± 457 vs. 3011 ± 1426, *p* = 0.001; 5000 ± 457 vs. 3010 ± 1413, *p* = 0.002) (Figure 1B).

### 2.2. Effects of Extracellular Vesicles on Collagen Production

**Figure 3:**
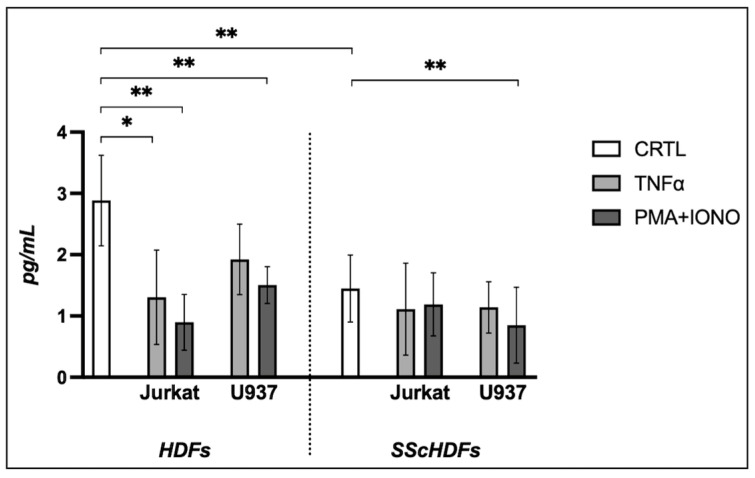
Evaluation of collagen production after stimulation with extracellular vesicles on fibroblasts. At baseline, the collagen production induced by HDFs and SScHDFs demonstrated a statistically significant difference. HDFs exhibited a notable difference from the control group when incubated with EVs derived from Jurkat cells stimulated with either TNFα or PMA + IONO. Similarly, EVs from U937 cells stimulated with PMA + IONO caused a significant difference. SScHDFs showed a significant reduction in collagen production only when incubated with EVs stimulated with PMA + IONO (* *p* < 0.05, ** *p* < 0.005). HDFs: human dermal fibroblasts; SScHDFs: scleroderma human dermal fibroblasts; EVs: extracellular vesicles; PMA: phorbol 12-myristate 13-acetate; IONO: ionomycin; TNFα: tumor necrosis factor-alpha.

The collagen production (reported in pg/mL) by the HDFs and SScHDFs (Figure 3) exhibited a statistically significant difference at baseline (2.885 ± 0.738 vs. 1.448 ± 0.545, *p* = 0.002). The HDFs showed a significant difference compared to controls when incubated with EVs derived from Jurkat cells stimulated with both TNFα (2.885 ± 0.738 vs. 1.306 ± 0.769, *p* = 0.014) and PMA + IONO (2.885 ± 0.738 vs. 0.897 ± 0.453, *p* = 0.005); likewise, incubation with EVs derived from U937 cells stimulated with PMA + IONO also showed a significant difference (2.885 ± 0.738 vs. 1.505 ± 0.299, *p* = 0.014). SScHDFs significantly decreased collagen production only when incubated with EVs stimulated with PMA + IONO (1.448 ± 0.545 vs. 0.849 ± 0.617, *p* = 0.034).

## 3. Discussion

This study examines the production of EVs by Jurkat and U937 cells and their distinct effects on fibroblast proliferation and collagen synthesis, highlighting their potential regulatory role in tissue remodeling.

EVs derived from PMA + IONO-stimulated Jurkat and U937 cells significantly reduced HDF proliferation in a dose-dependent manner, suggesting that these vesicles may carry bioactive molecules with inhibitory effects on fibroblast proliferation. Conversely, EVs from TNFα-stimulated cells had minimal impact on proliferation, except at the highest dose of U937-derived EVs, indicating that EV cargo composition varies depending on the stimulus, thereby influencing their functional impact on recipient cells [22,23]. In contrast, SScHDFs displayed a reduced responsiveness to EVs, potentially due to an intrinsic resistance to external proliferative stimuli, which is consistent with the altered fibroblast subpopulation dynamics observed in SSc [24].

One possible explanation for the diminished proliferative capacity of SScHDFs is that these fibroblasts may have already reached maximal proliferative potential due to chronic in vivo stimulation, leading to a state of exhaustion. Unlike HDFs, which retain the capacity to proliferate in response to stimuli, SScHDFs may exhibit proliferative limitations. Additionally, in vitro conditions may not fully replicate the in vivo environment, and non-immortalized fibroblasts may undergo mitotic senescence over successive divisions [25].

Collagen production assays further demonstrated that EVs modulate fibroblast function. PMA + IONO-stimulated Jurkat-derived EVs significantly reduced collagen secretion in both HDFs and SScHDFs, whereas TNFα-stimulated Jurkat EVs affected only HDFs. These findings suggest that PMA + IONO-stimulated EVs may carry inhibitory signals, possibly including microRNAs or cytokines [26,27]. U937-derived EVs had a more selective effect, significantly reducing collagen production in SScHDFs but not in HDFs, potentially reflecting differences in EV uptake or downstream signaling in different fibroblast subtypes.

An important factor influencing collagen production is the disease stage. Previous studies have demonstrated that fibroblasts from scleroderma patients at different disease stages exhibit distinct collagen production levels. In long-standing disease, fibroblasts already show the characteristic alterations associated with systemic sclerosis, including impaired collagen synthesis [28,29]. Moreover, the biopsy site appears to influence fibroblast behavior, as gene expression profiles differ significantly between fibroblasts from affected and unaffected skin. Those from affected areas display distinct transcriptomic signatures, potentially reflecting altered functional states [30,31,32].

Collagen production may also be modulated by cytokines such as IL-17A. Nakashima et al. [33] reported that IL-17A inhibits collagen production in fibroblasts from SSc patients, a process counteracted by endogenous TGF-β1 stimulation. Interestingly, fibroblasts from dcSSc patients do not respond to IL-17A due to the loss of IL-17AR expression, whereas fibroblasts from lcSSc patients retain sufficient receptor levels to mediate this inhibitory effect. Notably, IL-17F does not exert the same inhibitory effect, highlighting a specific role for IL-17A in collagen modulation.

T lymphocytes are the primary producers of IL-17, leading to the hypothesis that EVs derived from the Jurkat cell line, an immortalized T-cell model, may contain significant levels of IL-17. This could have contributed to the observed inhibition of collagen production. Although the IL-17A concentration within the EVs was not measured in this study, it remains a crucial variable for future investigation.

To quantify collagen production by fibroblasts, we employed an ELISA-based assay. While gene expression studies often indicate increased transcription of collagen-encoding sequences, this does not necessarily correlate with increased protein production. Consistent with Nakashima et al. [33], IL-17A likely inhibits collagen synthesis not through direct transcriptional repression but via the upregulation of miRNA-129-5p, which binds to and inhibits the translation of collagen mRNA.

## 4. Materials and Methods

### 4.1. Cell Cultures and Treatments

In this study, four cell lines were utilized: Jurkat (ThermoFisher Scientific/Gibco, Waltham, MA, USA), U937 (ThermoFisher Scientific/Gibco, Waltham, MA, USA), dermal fibroblasts isolated from skin biopsies of scleroderma patients (SScHDFs), and dermal fibroblasts isolated from healthy controls (HDFs). Cells were grown to confluence at 37 °C and 5% CO_2_ using DMEM (ThermoFisher Scientific/Gibco, Waltham, MA, USA) supplemented with 10% FBS (Sigma-Aldrich, St. Louis, MO, USA) and 1% penicillin–streptomycin (pen–strep; ThermoFisher Scientific/Gibco, Waltham, MA, USA). Cells were passaged using Accutase (Millipore, Billerica, MA, USA). The production of EVs by Jurkat and U937 cells (immortalized T lymphoblast and monocyte cell lines, respectively) was promoted using two different stimuli, TNFα (R&D Systems, McKinley Place NE, Minneapolis, MN, USA) and PMA (Sigma-Aldrich, St. Louis, MO, USA) + IONO (Sigma-Aldrich, St. Louis, MO, USA), resulting in four conditions: EVs from Jurkat cells stimulated with TNFα and PMA + IONO and EVs from U937 cells stimulated with TNFα and PMA + IONO. TNFα was reconstituted in deionized H_2_O at a concentration of 1 nM, while PMA and ionomycin were reconstituted in DMSO (Sigma-Aldrich, St. Louis, MO, USA) and used at concentrations of 10 ng/mL and 0.5 ng/mL, respectively.

### 4.2. Patients and Healthy Donors

Fibroblasts were obtained from skin biopsies of two healthy donors and four SSc patients, with written informed consent provided (Table 1). The healthy donor biopsies were collected at the General Surgery Unit of the University Hospital of Verona, while the SSc patient biopsies were obtained from the Autoimmune Diseases Unit at the same hospital. All patients conformed to the 2013 classification criteria for SSc outlined by the American College of Rheumatology (ACR) and the European League Against Rheumatism (EULAR) [34]. Patient classification into diffuse cutaneous and limited cutaneous SSc followed LeRoy’s criteria [35], which were revised in 2001 to include the early scleroderma diagnosis [36]. Fibroblasts were isolated from 0.6 cm forearm skin biopsies as detailed in our previous study [37].

### 4.3. Extracellular Vesicle Production and Isolation

Jurkat and U937 cells were stimulated with TNFα or PMA + IONO for 12 h; subsequently, they were centrifuged, and the supernatant was processed using polypropylene tubes. Following an additional centrifugation at 1500× *g* for 5 min, the supernatant was ultracentrifuged at 100,000× *g* for 20 min; this step is crucial for sedimenting the EVs. Two washes were then performed by ultracentrifugation, removing the supernatant and resuspending in APOP Buffer. The APOP Buffer was prepared with 5 mM KCl, 1 mM MgCl_2_, and 136 mM NaCl at a pH of 7.4 (all reagents from Sigma-Aldrich, St. Louis, MO, USA). An Optima XPN-80 Ultracentrifuge (Beckman Coulter, Brea, CA, USA) was used for these centrifugations. The EVs thus obtained were quantified in absolute numbers using a FACSCanto II flow cytometer (BD Biosciences, San Jose, CA, USA) with Perfect-Count Microspheres (Cytognos, Pol. La Serna, Santa Marta de Tormes, Salamanca, Spain).

### 4.4. Proliferation Assay

To assess the potential proliferative stimulus induced by EVs, 5000 fibroblasts (HDFs and SScHDFs) were plated and cultured in the absence of FBS. Subsequently, cells were stimulated for 24 h at 37 °C with three different quantities of EVs (absolute numbers): 12,500, 25,000 (the number of EVs used to stimulate our cells was derived from the study by Distler et al. [38]), and 50,000. Proliferation was evaluated using the Cell Counting Kit-8 (CCK-8; Sigma-Aldrich, St. Louis, MO, USA). Absorbance was measured using the TECAN Sunrise III plate reader (Tecan, Männedorf, Switzerland).

### 4.5. Collagen Production

As with the proliferation experiments, HDFs and SScHDFs were plated and cultured in the absence of FBS. A total of 40,000 cells were stimulated with 200,000 EVs [38] for 36 h. An evaluation of type 1 collagen was performed on the supernatant using an ELISA test (Cusabio Technology LLC, Houston, TX, USA).

### 4.6. Statistical Analysis

The data obtained were analyzed using GraphPad Prism 10 software (GraphPad Software Inc., La Jolla, CA, USA) for statistical analysis. All results are expressed as mean ± standard deviation (SD). The statistical relationships between the data were evaluated using the Mann–Whitney test. A *p*-value of <0.05 was considered to indicate statistical significance, signifying notable differences between the groups.

## 5. Conclusions

Our findings indicate that immune cell-derived EVs modulate fibroblast proliferation and extracellular matrix production, with effects dependent on the cell type and stimulus used for EV generation. The differential response of HDFs and SScHDFs highlights the importance of considering fibroblast heterogeneity in SSc pathogenesis. However, certain limitations must be acknowledged, including the use of immortalized cell lines instead of primary immune cells and the lack of distinction between lcSSc and dcSSc fibroblasts. Future studies employing larger cohorts and alternative sampling methods will be crucial to further delineate these findings and explore the therapeutic potential of targeting EV-mediated signaling in SSc.

## Figures and Tables

**Table 1:** Clinical characteristics of SSc patients and healthy donors.

	Sex	Age	Disease Form	Disease Duration
Healthy donors	1 Male 1 Female	32–45	-	-
Patients	1 Male 3 Female	34–51	2 dcSSc 2 lcSSc	3 < 5 years 1 > 5 years

## Data Availability

The raw data supporting the conclusions of this article will be made available by the authors without undue reservation.
